# Convergent evolution of modularity in metabolic networks through different community structures

**DOI:** 10.1186/1471-2148-12-181

**Published:** 2012-09-14

**Authors:** Wanding Zhou, Luay Nakhleh

**Affiliations:** 1 Department of Bioengineering, Rice University, Houston, TX, USA; 2 Department of Computer Science, Rice University, Houston, TX, USA

## Abstract

**Background:**

It has been reported that the modularity of metabolic networks of bacteria is closely related to the variability of their living habitats. However, given the dependency of the modularity score on the community structure, it remains unknown whether organisms achieve certain modularity via similar or different community structures.

**Results:**

In this work, we studied the relationship between similarities in modularity scores and similarities in community structures of the metabolic networks of 1021 species. Both similarities are then compared against the genetic distances. We revisited the association between modularity and variability of the microbial living environments and extended the analysis to other aspects of their life style such as temperature and oxygen requirements. We also tested both topological and biological intuition of the community structures identified and investigated the extent of their conservation with respect to the taxomony.

**Conclusions:**

We find that similar modularities are realized by different community structures. We find that such convergent evolution of modularity is closely associated with the number of (distinct) enzymes in the organism’s metabolome, a consequence of different life styles of the species. We find that the order of modularity is the same as the order of the number of the enzymes under the classification based on the temperature preference but not on the oxygen requirement. Besides, inspection of modularity-based communities reveals that these communities are graph-theoretically meaningful yet not reflective of specific biological functions. From an evolutionary perspective, we find that the community structures are conserved only at the level of kingdoms. Our results call for more investigation into the interplay between evolution and modularity: how evolution shapes modularity, and how modularity affects evolution (mainly in terms of fitness and evolvability). Further, our results call for exploring new measures of modularity and network communities that better correspond to functional categorizations.

## Background

**Figure 1:**
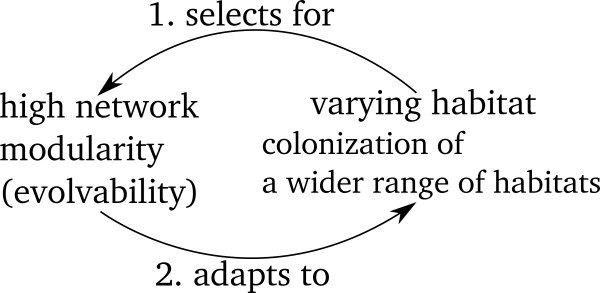
**A feedback loop between modularity and habitat variability.** Two different explanations of the association of the modularity score with the habitat variability.

Analyses of biological networks have revealed modular structures [1-5]. Parter et al. [6] found that bacterial species living in variable habitats have metabolic networks with significantly higher modularities than bacterial species living in less variable habitats. According to one explanation, since modularity promotes evolvability, enabling bacteria to quickly adapt to varying environments, having a more modular metabolic network is an evolutionarily favored trait for species living in open habitats such as soil and sea. In other words, high modularity is selected for by evolution for species living in these varying habitats (edge 1 in Figure 1). The robustness of metabolic networks, a concept related to modularity [7], as measured by the maintenance of a phenotype (e.g., growth) under perturbation (e.g., mutation or gene loss), has been shown, both *in vivo* and in simulation, to have risen from fluctuating environments [8,9]. An alternative explanation can be formulated from the other direction: because species with a higher modularity in their metabolic networks are more capable of adapting to changes in environment, they colonize a wider range of habitats, giving rise to the observation that bacteria living in varying habitats have more modular metabolic networks (edge 2 in Figure 1). In another recent study of an Archaea data set [10], such relationship between modularity and habitat variability was not found, which calls for more investigation of alternative explanations.

Modularity as a graph-theoretic concept, when studied on biological networks, can be quantified in different ways [6,11-15]. In the works of Parter et al. [6] and Kreimer et al. [15], modularity is based on the definition of Newman and Girvan [16]. This definition quantifies the extent to which the graph connectivity of a network exhibits a modular structure, that is, communities with a majority of the connections falling within, rather than across, communities. Roughly speaking, the modularity score *Q*[16] (see Methods), which is a quantity associated with a partition of the network, indicates how much more likely it is for an edge to be placed inside a community from that partition than would be expected from a random selection of neighbors for a node of a certain degree. The partition of nodes that gives rise to the maximum *Q* value is regarded as the community structure of the graph, and the score itself is taken to be the graph’s modularity.

Although the modularity score depends on the community structure, similar modularity scores may arise from different community structures. It is natural to ask (and is currently unknown) whether a specific modularity (high or low) of metabolic networks is the result of acquiring a similar community structure or of achieving different community structures. More specifically, assuming that network modularity plays an adaptive role [17], as is the case for the first explanation (Figure 1), is it the modularity score that confers higher fitness regardless of the community structure giving rise to it, or is it the community structure that is the unit of selection and modularity is conserved only as a consequence? If modularity is achieved via similar community structures, it might be the community structure that is the unit of selection under different environments. That said, any observed association of modularities with the environmental features [6,15] or growth conditions [10] would naturally give rise to a question as to whether such a correlation arises due to similar community structures (which, by definition, would have similar modularity scores) or different community structures with similar modularity scores.

In this work, we analyzed metabolic networks of species spanning three kingdoms of life by computing their community structures and modularity scores (see Methods for details on metabolic network reconstruction). We compared the difference in community structures against the difference in modularities and the genetic distance, to investigate the correlation, or lack thereof, among the three. The results suggest that the difference in community structures does not parallel the difference in modularity scores we compute, except when community structures are extremely similar. That is, we find that larger community structure differences do not necessarily mean larger differences in modularity scores and vice versa, which is an indication of convergent evolution of modularities via different underlying community structures. To further understand the evolutionary driving force behind such convergent evolution, we revisited the analysis of Parter et al. [6], which first associated modularity with habitat variability, but under different aspects of the microbial life styles, including temperature preference and oxygen requirement. We also confirmed the finding of Kreimer et al. [15] that the size of the metabolome (the number of enzymes) is a major determinant of the modularity score, even after the score is normalized and believed to be size-independent on general (non-metabolic) networks.

From a computational perspective, a contribution of this paper is an improved heuristic based on spectral decomposition for modularity optimization [18] using a self-organizational merge and resplit refinement. The goal of this improvement is to deterministically identify more optimal modularity scores and community structures efficiently. We show, on well-studied benchmark data sets, that compared to the original algorithm of Newman [18] and some other existing algorithms [16,19-21], our algorithm achieves higher *Q* scores at the cost of only a moderate increase in time.

## Results

### Community structure differences do not parallel the modularity differences

Previous studies have shown the association of modularity of metabolic networks with variability of the living environment of species [6] and the bacterial life style [15]. However, it remains unclear whether or not this association is a consequence of any further association with the underlying community structure. In other words, the relation between the living environment and modularity might be a consequence of the habitats’ association with the community structure. To answer this question, we investigate whether for a similar modularity score there exist multiple distinct community structures in metabolic networks of different species.

**Figure 2:**
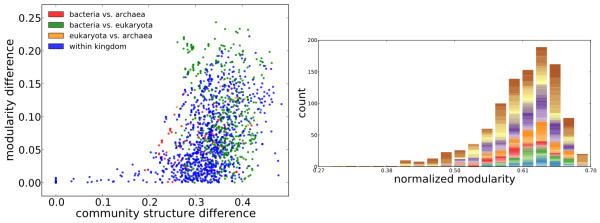
**Community structure vs. modularity.** Left) Community structure difference vs. modularity difference: Difference in community structure is computed by 1−*MI *where *MI* is the mutual information between the two community structures. Right) Distribution of modularity scores colored by the cluster to which the community structures of the metabolic network belongs (See Methods for the method used to cluster species based on the distance in the community structures). Modularity scores are normalized with respect to scores based on randomized networks (See Methods). The normalized modularity is believed to have network size-dependent factors removed, allowing networks of different sizes and connectivity to be comparable in modularity [22]. Each color corresponds to a community structure cluster. The height of the bar (or bar segment) is proportional to the number of species in each cluster falling into the particular bin of modularity scores.

The results in the left panel of Figure 2 show that a smaller difference in modularity is not an indication of more similar community structures.

When the community structures are similar (roughly < 0.2), their modularity scores must be similar. Such dependency is expected from the definition of modularity. Beyond 0.2 in the difference of community structures, modularities vary significantly, from very similar to very different, despite different community structures. In other words, the same modularity score may be achieved via different community structures. Such convergence at the modularity level takes place mostly between bacteria and eukaryota, though also happening between species within the same kingdom, as indicated by the green and blue dots on the bottom right corner of the left panel of Figure 2. To further explore this relationship between modularity scores and community structures on metabolic networks, we plotted the distribution of modularity scores for each community structure cluster (Figure 2) obtained through hierarchical clustering (see Methods). In the right panel of Figure 2, we see that most community structure clusters span many bins of modularities and for each bin of modularity scores, community structures from different clusters can be discerned. This indicates that similar modularity scores found on metabolic networks can stem from different community structures.

### Convergent evolution of modularity scores

**Figure 3:**
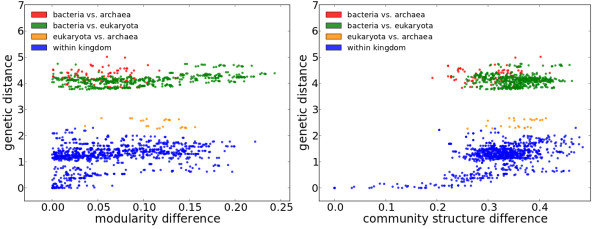
**Difference in modularities (left) and community structures (right) vs. genetic distance (in substitutions per site).** The gap in the middle of the plots corresponds roughly to the long branches separating bacteria from the rest (archaea are closer to eukaryota than to bacteria and bacteria are roughly as close to archaea as to eukaryota).

To investigate the evolution of modularity scores and community structures, we plotted for every pair of species the difference in their modularity scores and community structures against their genetic distances (see Methods for the computation of the genetic distances); results are in Figure 3. In the left panel of Figure 3, modularity difference can be close to zero even between species across the kingdoms, which supports the hypothesis of convergent evolution of modularity. On the contrary, community structures are similar only when two species are genetically very close (see the right panel of Figure 3). Since closely-related organisms have similar enzyme profiles (see Additional file 1: Figure S1) which result in similar metabolic networks’ connectivity, and enzyme profile similarities are negatively correlated with community structure differences (Additional file 1: Figure S2), it makes sense that closely-related organisms also have similar community structures.

### Convergent evolution of modularity is driven by life style

Knowing that similar modularity may be achieved independently via different community structures, we revisit the question of what drives the convergent evolution of modularity. We studied several factors ranging from the size of the metabolome (the number of enzymes and the size of the network under the current choice of network semantics) to environmental factors that include temperature preferences and oxygen requirements.

#### Network size remains a determinant of normalized modularity on enzyme networks

**Figure 4:**
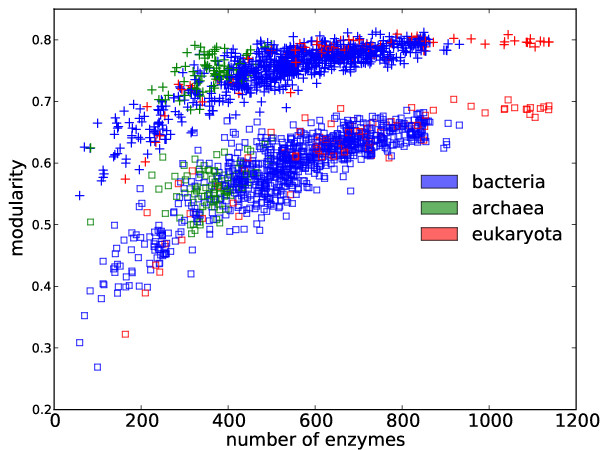
**Modularity vs. the number of enzymes.** The numbers of enzymes are significantly correlated with modularity scores (“+” markers). Such observation remains after modularity scores are normalized (square markers).

Network size is reported to be an important determinant of network modularity [15]. We show that: although the normalized modularity is believed to be independent of the network size [22], dependence remains for normalized modularities in the case of enzyme networks (see Methods). In Figure 4, we plot the modularity scores and the number of enzymes. We observe that modularity is significantly correlated with the number of enzymes, whether modularity is normalized or not (Spearman’s ranked *r *= 0.85,*p *= 2.0 × 10^−282^ in the normalized case and *r *= 0.80,*p *= 2.6 × 10^−229^ in the unnormalized case). We also see that species with a reduced metabolome (such as those under the clade of Mollicutes and Rickettsiales) possess smaller modularities in their metabolic networks (see Discussion), which is consistent with our observation here. The dependence of modularity on the number of enzymes is sensitive to rewiring (see Additional file 1: Figure S3). It is worth mentioning that similar correlation is seen on: 1) synthetic linear graphs (graphs composed of nodes linearly concatenating each other); see Additional file 1: Figures S4; and S2) the line graph transformations [23] of rewired compound networks with currency metabolites deleted; see Additional file 1: Figure S5, implying that their resemblance to the organization of metabolic networks may explain the dependence of Newman’s modularity on the sizes of the network.

#### The association of the environmental variability with the modularity is a consequence of its association with the number of enzymes

**Figure 5:**
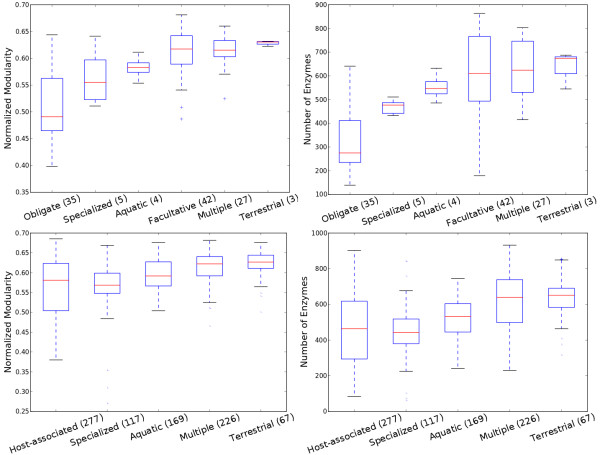
**Environment variability and modularity.** (Top row) On a small data set of 116 bacteria, habitat variability vs. normalized modularity (left) (Kruskal-Wallis H-test *p *= 5.48 × 10^−11^) and habitat variability vs. the number of enzymes (right) (Kruskal-Wallis H-test *p *= 1.03 × 10^−10^). (Bottom row) On a large data set of 806 microbes, habitat variability vs. normalized modularity (left) (Kruskal-Wallis H-test *p*=6.51×10^−26^) habitat variability vs. the number of enzymes (Kruskal-Wallis H-test *p *= 1.03 × 10^−30^).

When revisiting the association of modularity to environmental variability, we find a similar trend as is reported by Parter et al. [6] (left column of Figure 5, with the data set used in [6] plotted in the top row and a larger data set plotted in the bottom row). However, an identical trend is also seen for the number of enzymes (right column of Figure 5). This means that the association of modularity with the environmental variability might be a consequence of the difference in the numbers of enzymes between species living in environments of different variability, given the aforementioned strong correlation between modularity and the number of enzymes. In the study by Parter et al. [6], the category “host associated” in the classification from NCBI was further refined into “obligate” and “facultative” to differentiate bacteria that are able to survive without the host from those that cannot. We find that under this refinement, obligate species have a significantly smaller number of enzymes than facultative ones (one tailed Wilcoxon rank-sum test *p *= 4.6 × 10^−10^). Moreover, this refinement is not perfect (for example, the smallest facultative species *B. burgdorferi* is often described as obligate [24,25] and the second largest obligate species *R. Baltica* in the data set is in fact free-living marine bacteria [26]). Therefore, the difference in the number of enzymes between facultative species and obligate species could in fact be more striking.

It is conceivable that microbes capable of coping with a varying and open habitats have a larger metabolome and microbes that lead specialized lifestyles have a smaller metabolome. An extreme case is that bacteria leading an obligate lifestyle has a reduced metabolome. One explanation of this phenomenon is that unnecessary genes for living in a specialized niche that only increase the overhead of maintenance were lost during evolutionary history [27-30]. For example, the *γ*-proteobacteria *B. aphidicola* lack the genes for the synthesis of tryptophan, riboflavin, fatty acids and phospholipids due to its endosymbiosis with aphids [31,32]. Here we see that the numbers of enzymes of 8 insect endosymbionts in *γ*-proteobacteria are significantly smaller than the other species in our dataset (one-tailed Wilcoxon rank-sum test *p *= 1.1 × 10^−6^). Even the largest of these endosymbionts (*B. pennsylvanicus*, 366 enzymes) has a smaller metabolome than the smallest non-endosymbiont (*D. nodosus*, 459 enzymes). Modularity scores of endosymbionts are also significantly smaller than non-endosymbionts (one-tailed Wilcoxon rank-sum test, *p *= 2.5 × 10^−6^).

To study whether habitat variability truly affects the modularity of the metabolic networks besides the effect of the number of enzymes, we binned the species into groups with the number of enzymes in bins ranging within at most 50 enzymes. Out of 24 bins from 100 to 820 with the number of enzymes incrementing by 30, 16 bins contain at least two categories of species each of which has more than 10 members. Only in 4 of these 16 bins (310∼340, 430∼460, 490∼520, 520∼550) habitat variability significantly (Kruskal-Wallis H-test *p *< 0.05) affects the network modularity. This fact shows that most of the seeming dependence of modularity on the habitat variability may disappear if the number of enzymes is controlled.

#### The order of modularity is the same as the order of the number of enzymes under the classification based on temperature preference but not oxygen requirement

**Figure 6:**
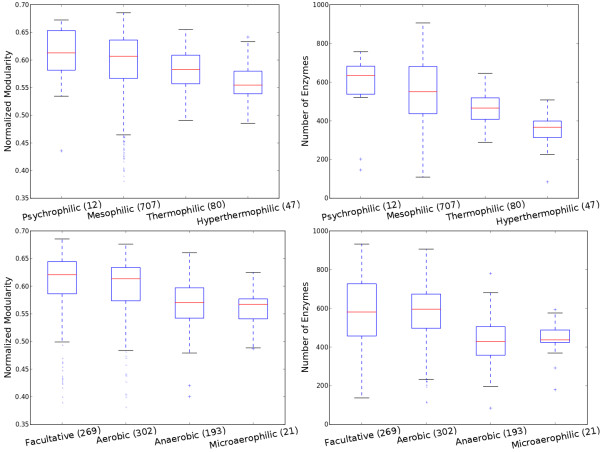
**Modularity and environment factors.** (Upper left) normalized modularity vs. oxygen requirement (Kruskal-Wallis H-test *p *= 2.96 × 10^−25^); (Upper right) number of enzymes vs. oxygen requirement (Kruskal-Wallis H-test *p *= 3.35 × 10^−33^); (Lower left) normalized modularity vs. temperature requirement (Kruskal-Wallis H-test *p *= 5.52 × 10^−9^); (Lower right) number of enzymes vs. temperature requirement (Kruskal-Wallis H-test *p *= 1.06 × 10^−19^).

Temperature preferences and oxygen requirements can be more objective measures of environmental variabilities. By comparing the modularities against the temperature (top row of Figure 6), we find that thermophilic and hyperthermophilic bacteria have a lower modularity (see Additional file 1: Table S1 for pairwise comparison). In all the cases where we compare modularity, we also compare the number of enzymes from different categories. We observed a significant difference in every case. And the number of enzymes has a consistent trend as modularity, which again indicates that the association of modularity to the temperature is mediated by the number of enzymes. The variation in the number of enzymes can be understood recognizing the biochemical fact that only a small amount of enzymes can function properly under elevated temperature.

By comparing the modularities against the oxygen requirements of the species (bottom row of Figure 6), we find that facultative bacteria have the highest modularity. Microaerophilic bacteria have the least modularity. Facultative bacteria are ones that normally utilize oxygen as their electron receptor but can also ferment other endogenous electron receptors such as ethanol and lactate. On the contrary, microaerophiles have the most strict requirement for oxygen. For them, oxygen is not only a requirement for survival, but the concentration of oxygen must also be lower than what is present in the atmosphere. If environmental variability should explain the difference in modularity, the flexibility in oxygen usage, as one way of reflecting environmental variability, supports such explanation: facultative bacteria have higher modularity than strictly aerobic and strictly anaerobic bacteria. And strictly aerobic bacteria have higher modularity than microaerophiles. There is no significant difference in modularity between anaerobic bacteria and microaerophiles (two tailed Wilcoxon rank-sum test *p *= 0.40, same result for the number of enzymes, *p *= 0.57). However, bacteria that are capable of freely metabolizing oxygen (facultative joined with aerobic) have significantly (one tailed Wilcoxon rank-sum test *p *= 5.5 × 10^−26^) higher modularities than those who have limited capability of handling oxygen or have to rely on fermentation (microaerophiles joined with anaerobic). The same result is obtained when the number of enzymes are compared (*p *= 1.1 × 10^−35^). Comparison between only strictly aerobic microbes against strictly anaerobic microbes also indicates statistical significance (one tailed Wilcoxon rank-sum test *p *= 6.9 × 10^−16^ in modularities and *p *= 1.2 × 10^−30^ in the numbers of enzymes). Facultative bacteria have significantly higher modularities than strictly aerobic bacteria (one tailed Wilcoxon rank-sum test *p *= 0.0025). However, a null hypothesis is accepted when it comes to the number of enzymes (one tailed Wilcoxon rank-sum test *p *= 0.18), meaning that the significant difference in modularity between facultative bacteria and strictly aerobic bacteria is not a consequence of the difference in the numbers of the enzymes.

## Discussion

### Modularity-based communities are topologically meaningful yet do not reflect biological functional classifications

Despite the existing studies on the modularity of metabolic networks and reported limitation in modularity-based community detection such as the resolution limit [33] (optimizing the modularity score might fail to detect small communities), the non-locality [34] (the local delineation of a community depends on the global network connectivity) and the extreme degeneracy [35] (there might exist multiple optimal/suboptimal community structures), it remains unclear whether, in this specific case of metabolic networks, modularity-based communties reflect the graph-theoretic intuition of a community structure.

**Figure 7:**
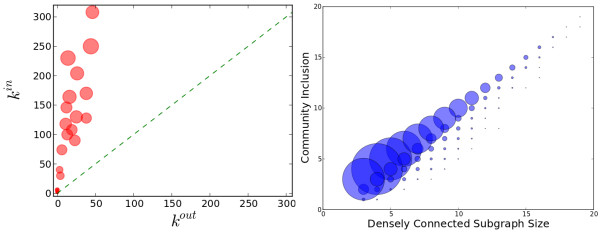
**Topological meaning of communities detected.** (Left) Following [36], *k*^*in *^is the intra-community degree and *k*^*out *^is the inter-community degree of each node in the metabolic network of *E.coli*. Summation ( $\sum k^{\text{in}}$ and $\sum k^{\text{out}}$) of intra and inter community degree is over all nodes in each community. The size of the circle is proportional to the size of the community. As is shown, all the communities have $\sum k^{\text{in}}>\sum k^{\text{out}}$ and most nodes are have *k*^*in *^>* k*^*out*^(Additional file 1: Figure S6). (Right) Inclusion of densely connected subgraphs in the community. The center of each circle corresponds to an observation of a densely connected subgraph *K* (in any of the 1021 species investigated). X-axis indicates the size of *K* and Y-axis indicates the maximum overlap of *K* with a community among all the communities detected from the metabolic network of the same species. The size of the circle is proportional to the number of instances that give the observation.

To briefly investigate whether the modularity score (and the corresponding community structures) reflects the intuitive concept of being “modular” (that is, whether a graph with high modularity score can indeed be partitioned into dense subgraphs with sparse connectivity across subgraphs) given the specific topologies of metabolic networks, we compare the communities based on Newman’s definition against one of the many other definitions, namely the one by Radicchi et al. [36], where the community structure definition in strong sense requires that for all the nodes in the network, the number of neighbors of the node from the same community (*k*^*in*^) be greater than the number of neighbors of the node from different communities (*k*^*out*^). The definition in a weaker sense only require the sum of *k*^*in*^ be greater than the sum of the *k*^*out*^ over all nodes in a community. We computed the *k*^*in*^, *k*^*out*^ for all the nodes in the metabolic network of *E.coli*. We find that the partitions obtained via modularity optimization satisfy the weaker definition (see Figure 7). Most communities also satisfy the strong definition (Additional file 1: Figure S6). In all the 10 nodes in *E.coli* that break the definition in the strong sense, the connections to nodes from the same community outnumber the connections to any one of the other communities to which the node does not belong (even though the sum of outward connections is greater). This explains why these nodes are not classified into any of the other communties. These 10 nodes consist of 2 oxidoreductase, 6 transferase and 2 lyases. No particular preferences of pathway participation from these exceptions was observed.

In order to test the extent of the resolution limit of the modularity based community detection on metabolic networks, we computed densely connected subgraphs using the SIDES program [37]. As shown in the right panel of Figure 7, most of the densely connected subgraphs are contained in the same communities, which is a rough indication of the exemption from the resolution limit.

Despite these findings, the definition of modularity we use might still be problematic when applied to linear/sparse graphs. As we show in Additional file 1: Figure S4, the longer the linear graph, the higher its modularity, which is problematic given that two line graphs should be intuitively considered equally modular regardless of their lengths.

Another crucial question in studying the modularity of metabolic networks is whether the communities detected carry any functional meaning (in the biological sense). Intuitively, modularity or density-based methods would not identify linear, or more generally sparse, pathways. To answer this question, we investigate the functional meaning of communities computed on the metabolic network of *E.coli*. We find that these communities have limited specificity to partitions based on biological functions.

**Figure 8:**
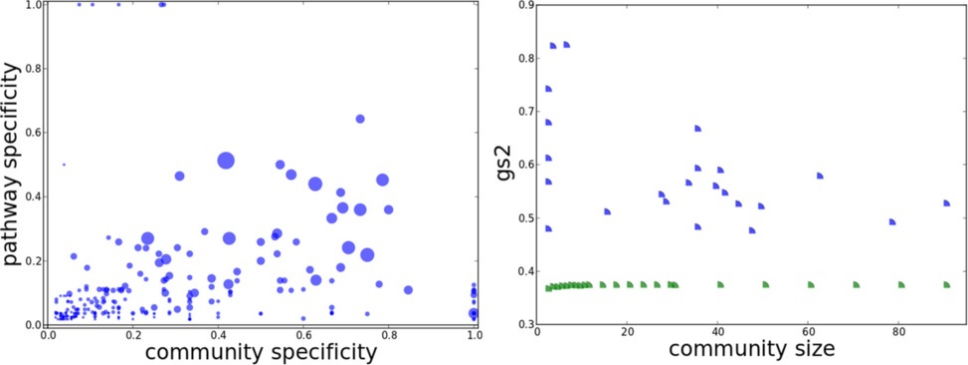
**Biological meaning of communities detected.** (Left) The community-pathway overlap in E. coli’s metabolic network. Each point corresponds to a community-pathway pair, where the size of a point reflects the size of the overlap between the community and pathway. Similar results are observed on compound networks with currency metabolites deleted (Additional file 1: Figure S7). (Right) Gene Ontology Enrichment of Communities. The similarity of genes inside each community detected from *E.coli* (blue) against the similarity of genes randomly selected (green).

First, we explore how communities overlap with established biochemical pathways. Second, we explore the functional similarity based on the Gene Ontology (GO) [38]. For correlation with biochemical pathways, we computed for each pair of community-pathway the community-wise and pathway-wise specificities, defined as the number of reactions shared by both the community and pathway and normalized by the size of the community and size of the pathway respectively. Based on these definitions, if a community is completely contained within a pathway, its community-wise specificity (with respect to that pathway) is 1, and if a pathway is completely contained within a community, its pathway-wise specificity (with respect to that community) is 1. We computed these two specificity measures by using the biochemical pathways of E. coli obtained from the KEGG database [39] (left panel of Figure 8). Three patterns are worth observing in this figure. The top right corner has no points, an indication that there is no 1-1 correspondence between pathways and communities. This conforms to our intuition that biochemical pathways are very sparse graphs, whereas communities correspond, roughly, to dense subgraphs. Second, the bottom left corner is very dense, further supporting the lack of a 1-1 correspondence; however, it is important to notice that the points in this corner are all small, reflecting very small overlap between pathways and communities. Third, the pathways and communities with high specificities have relatively large overlaps. These three trends combined indicate that a few pathways are between 50%-80% contained within communities, very few communities are contained within pathways (see Additional file 2 for a list of representative cases) and the majority of pathways are fragmented across communities.

We studied the Gene Ontology (GO) annotation of the genes that transcribe the enzymes in the *E.coli* network using GS^2^[40], a measure that quantifies the similarity of GO terms among a group of genes. In order to tell whether enzymes inside the same community have a similar ontology, we ran GS^2^ on genes that are annotated to transcribe enzymes belonging to the same community. We find that genes inside the same community have a higher similarity of GO annotations than the same number of genes but randomly selected from the gene pool of the organism (right panel of Figure 8). Following Bauer et al. [41], we test whether a community is functionally significant by whether there is a significant enrichment of any GO term. The GO specificity is calculated by dividing the extent of overlap between the GO term and the community by the total number of genes that have that GO term in *E.coli*. The community specificity is calculated by dividing the extent of overlap between the GO term with the community by the number of genes that transcribe the enzymes in the community. GO-community pairs where the GO term significantly annotates the community are isolated (tested against the hypergeometric distribution with Bonferroni correction for multiple comparisons, *α *= 4.7 × 10^−5^[42]). In spite of many GO-community annotations with significant p-values, no clear 1-1 correspondence between GO terms and community structures is seen (Additional file 1: Figure S8). This suggests that the GO similarity among genes inside the same community might result from their closer distance on the network, assuming genes inside a community are closer on the network and nodes closer on the network are more likely to share GO annotations.

### Community structures are only kingdom-specific

**Figure 9:**
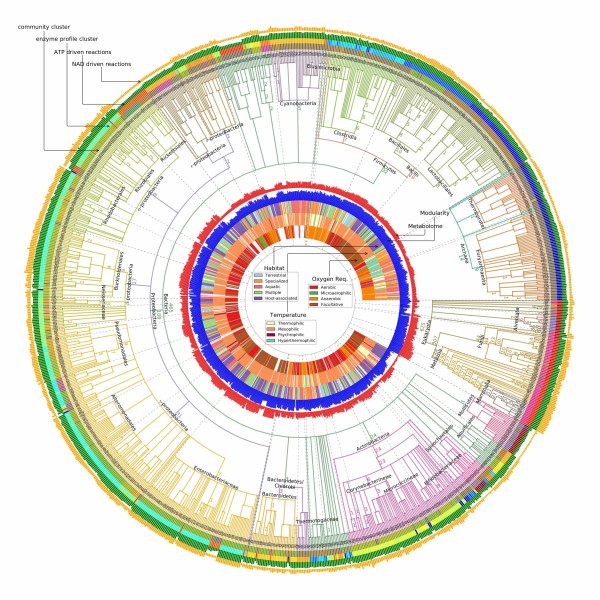
**Clustering of community structures.** The outermost track is colored according to the clustering of community structure. The phylogeny and names of the clades are obtained from the NCBI taxonomy. The blue track corresponds to the normalized modularity score (very similar pattern has been observed in unnormalized modularity scores, omitted due to page limit). Enzyme clusters are obtained by flattening the UPGMA linkage such that the cophenetic distance among leaves in each cluster is less than 0.5. The red track indicates the number of annotated enzymes in each species.

By comparing community structures of the networks across multiple species, we find that community structures are only specific at the kingdom level but not lower. Clustering of species based on the mutual information of community structures separates species from different kingdoms with some exceptions, as is shown in Figure 9. The discrimination of kingdoms from the community structure of metabolic networks is brought about by the similarity of *enzyme profiles*, or the spectra of all enzymatic activities as are characterized by the sets of Enzyme Commission (EC) numbers, among species from the same kingdom. As is shown in Figure 9 where we label on each branch the number of enzymes appearing exclusively in the descendants of the branch (an indication of metabolic innovation specific to the lineage), both bacteria and eukaryotes have their characteristic metabolic capabilities (465 and 625 respectively) while archea tend to share their metabolic capabilities with species from other kingdoms (14 unique enzymes).

Due to the independence of enzyme-reaction relationship from the choice of the species, enzyme profiles directly determine the connectivity, and hence the community structure of the metabolic networks. Any difference in the community structure is a result of some difference in the enzyme profile. To see whether different enzyme profiles would generate similar community structures, we cluster the species by their enzyme profiles using Unweighted Pair Group Method with Arithmetic Mean (UPGMA) [43]. We find that the clusters based on enzyme profiles agree to a substantial degree to the clusters based on community structures (third and fourth tracks from the outer rim in Figure 9).

## Conclusions and prospects

In this paper, we conducted an evolutionary analysis of metabolic network modularity in order to explore whether it is the network modularity or the community structure on which the modularity score is based, is the unit of selection. We showed that modularities undergo convergent evolution via different community structures. Further we revisited the association of the modularity score to environmental variability and extended the analysis to other aspects of microbial life styles. We found that on enzyme networks, the number of enzymes, which is also the size of the network and could also indicate the size of the metabolome, might be a determinant of the observed association between modularity and environmental variability. Further, we identified a strong association between network modularity and the microbe’s temperature and oxygen requirements. We also found that modularity-based community structure does not correspond to biological functional classifications and is conserved only at the kingdom level.

An important confounding factor with metabolic network analysis is the network semantics, or what the nodes of the network represent and how the network is reconstructed. Previous studies have been based on different reconstructions and network semantics; for example, Parter et al. [6] considered networks with nodes representing metabolites while Kreimer et al. [15] considered networks with nodes representing enzymes. In order for the results to be comparable, we considered in this work four different alternatives (see Data). We found that the same analysis on different network reconstructions can lead to qualitatively different conclusions. For example, the correlation of modularity to the number of enzymes is only true for enzyme networks (Figure 4 and Additional file 1: Figure S9) but not for compound networks (Additional file 1: Figure S10 and Figure S11). For compound networks, we find a significant difference in normalized modularity among different groups but no clear association between modularity and habitat variability (Additional file 1: Figures S12 and S13) in contrast with enzyme networks (Additional file 1: Figure S14). We cannot repeat the association of network modularity to the environmental variability on compound network with currency metabolites deleted, as reported in Parter et. al. [6]. Our result is consistent with a more recent analysis on an Archaean data set where no association was found either [10]. Discrepancy might result from the differences in the network reconstruction, algorithm used to optimize modularity or data used (due to different database releases). Despite different network semantics, it remains consistent that normalized modularity is significantly different among the groups classified by temperature requirements while not as significantly different among the groups classified by the oxygen requirements (Additional file 1: Figures S15, S16 and S17) and that modularity scores are achieved via distinct community structures (Additional file 1: Figure S18, S19 and S20).

Our work calls for more biologically meaningful definitions of the modularity for metabolic networks. Modules under such definition might not be graph-theoretically intuitive. Density-based definitions do not describe well pathways and sparse graphs which seem to be ubiquitous in biological systems (e.g., a biochemical pathway may be very sparse and does not fit the definition of a graph-theoretic module). Another drawback from defining modularity as a graph-theoretic concept in metabolic networks is that metabolic systems are inherently hypergraphs instead of standard graphs [44]. Adopting the graph-theoretic definition of modularity imposes a graph representation onto the metabolic system. Thus our work also calls for more careful scrutiny on the recent results related to the adaptive roles on modularity scores and their association with biological phenotypes. Adaptive roles should be explained under specific network reconstruction and care should be taken when one makes generalized conclusions.

## Methods

### Community detection and modularity

The modularity score of a network is defined as follows [16]: consider a network with its set of nodes *V * and set of edges *E*, the *Q* score is defined as a function of a partition P of *V *,

(1) $$Q(P)=\underset{i}{\sum }(e_{\text{ii}}-a_{i}^{2})$$

where *e*_*ii *_is the fraction of edges in community *i* (over all edges in the network) and *a*_*i*_ is the fraction of edges that are incident on a node in community *i*. The highest *Q* score attained over all possible partitions, $\arg\underset{P}{\max}Q(P)$, is defined as the network’s modularity. Two communities are neighbors if there is an edge connecting any pair of their members, i.e., *C*_*i*_ is a neighbor of *C*_*j*_ if there is some *p*∈*C*_*i*_ and *q*∈*C*_*j*_ such that (*pq*)∈*E*. Several algorithms have been devised to estimate the modularity together with its corresponding community structure; see [45] for a review. In this work, we improve the algorithm of Newman [18] to optimize the modularity score. The improvement is achieved by global merge and resplit and is given in Algorithm 1.

#### Algorithm 1

Merge-Resplit

**Input** : Graph *g*=(*V*,*E*).

**Output**: A partition Pto maximize *Q*. 

1. P= RecursiveBipart(*V*,*E*);

2. **do**

3. **for**$C_{i},C_{j}=\text{neighbors in}\;P$**do**

4. $C_{\text{merge}}=C_{i}\cup C_{j};$

5. $P^{\prime }$ = RecursiveBipart(*C*_*merge*_,*E*);

6. **foreach***v*∈*C*_*merge*_**do**

7. $S(v)=\left\{\begin{array}{rl} 1 & \text{if}v\in C_{i}\\ -1 & \text{if}v\in C_{j} \end{array}\right);$

end

8. $P^{\prime \prime }$ = KirnighanLin(*C*_*merge*_,*E*,*S*);

9. $P={\text{argmax}}_{P\in \{P^{\prime },P^{\prime \prime }\}}Q(P);$**end**

**while**Pis varying;

10. **return**P

Procedure RecursiveBipart on line 1 and 5 follows Newman [18] which recursively bipartitions its input graph using spectral decomposition by [46,47], with the KirnighanLin (on line 8) procedure interleaved on each level of bipartitioning. Following Newman [18], given any bipartion (*C*_*i*_, *C*_*j*_), if we define Q as a quadratic product of graph Laplacian *L* and the membership vector *S* (as defined in line 6).

(2) $$Q=\frac{1}{2}S^{T}\text{LS}$$

Optimal Q is achieved by finding *S* with the leading eigenvalue of *L*. Eigen problems are solved using shifted power method. Each step in KirnighanLin procedure both on line 8 and inside RecursiveBipart (following Newman [18]) optimizes the boundary of two communities by greedily swapping a pair of nodes whose exchange results in the largest increase in *Q*. The intermediate state with the highest *Q* is returned.

After the initial decomposition from RecursiveBipart, each pair of communities thus obtained are merged and fed again into RecursiveBipart, whose spectral property guarantees that the computed partition, which might contain one, two or more subsets, yields no lower *Q*. The new partition obtained is compared with a partition obtained by directly applying the KirnighanLin procedure to the boundary between the two original communities. The partition that gives rise to the larger *Q* is kept. This is to ensure the new partition will lead to better optimization than the current one. Such merge-resplit process continues until the partition no longer varies after completely traversing the boundaries between all pairs of the neighboring communities, thereby reaching a self-organized state (a state in which boundaries between any two neighboring communities can not be further improved). The modified algorithm outperforms the existing deterministic algorithms and some computationally heavy stochastic methods, in maximizing *Q*, as is shown in Additional file 1: Table S2 (see Additional file 1: Table S3 for the computation time at each benchmark data set). A C implementation of the improved algorithm is available at http://www.bioinfo.cs.rice.edu/.

#### Normalized modularity

Following Parter et al. [6], normalized modularity is defined as

(3) $$\frac{Q-Q_{\mathrm{rand}}}{1-1/M-Q_{\mathrm{rand}}}.$$

where *M* is the number of communities in the real network and *Q*_rand_ is the mean Q value of randomized networks. To determine the number of rewiring operations in computing *Q*_rand_, we use the leveling of global clustering coefficient [48] of the network as the signal for convergence. For each edge semantics, the number of rewiring operations required to make level the global clustering coefficient of the largest network is used for all species when we rewire its metabolic network of the particular edge semantics (see Additional file 1: Figure S21). Each rewiring operation involves swapping the ends of two randomly chosen edges. This process keeps the networks’ degree distribution. Alternative null models can involve the constraint of the number of short cycles. We do not consider the constraint due to difficulty in identifying all the cycles and ambiguity in determining the length of the cycles constrained.

### Mutual information

Given two partitions Aand B (in this work, Aand Bare the community structures of networks from two different species), the mutual information MI(A,B)[49] is defined as,

(4) $$\frac{2\times (H(A)+H(B)-H(AB))}{H(A)+H(B)},$$

where the marginal entropy is defined as,

(5) $$H(A)=\underset{i\in A}{\sum }\frac{N_{i}}{N}\text{log}(\frac{N_{i}}{N}),$$

*N*_*i*_ is the number of nodes that belong to set i∈A and *N* is the total number of nodes common to both networks. The joint entropy is defined as,

(6) $$H(AB)=\underset{i\in A}{\sum }\underset{j\in B}{\sum }\frac{N_{\text{ij}}}{N}\text{log}(\frac{N_{\text{ij}}}{N}).$$

and *N*_*ij*_ is the number of nodes that belong to both set i∈A and set j∈B.

### Clustering of community structures

We cluster the community structures by using hierarchical clustering (nearest point algorithm) implemented in the open source SciPy [50] package. The distance between any two networks is 1−*MI* where *MI* is the mutual information between their community structures. Clusters are flattened by looking for largest sets of individuals such that the pairwise distance among its members are within a chosen threshold based on inspection. The threshold used is 0.7. The clusters of species by community structure similarity are listed in Additional file 3.

### Data

We obtained manually annotated metabolic networks of 1021 species from the KEGG database [39] (see Additional file 1: Figure S22 for a summary of enzymatic annotations and Additional file 4 for a summary of organisms). The networks were assembled following Kreimer et al. [15]. Reaction direction information was extracted from the pathway KGML file provided by KEGG. Altogether there are 3548 KEGG reactions with direction identified, leaving 4635 reactions denoted as reversible. From these data, we assembled four types of networks using four different semantics, namely, compound networks where nodes are metabolites, enzyme networks where nodes are enzymes, compound networks with currency deletion where nodes are metabolites and connections are pruned as in [51,52], and enzyme networks with currency link deletion where nodes are enzymes and connections are pruned as in [15]. Analyses shown in this work are of enzyme networks with currency link deletion unless stated otherwise. The species’ habitat variability, temperature preferences and oxygen requirements are obtained from NCBI Genome Project Organisms Info Tab ( http://www.ncbi.nlm.nih.gov/genomes/lproks.cgi).

To conduct an evolutionary analysis of the data, we make use of the phylogeny, both branching pattern and branch lengths with branch lengths measuring sequence divergence in the unit of the number of subtitutions per site, inferred by [53]. Out of the 1021 species, only 56 appear in this phylogeny. Therefore, when we compare the community structures and modularity scores against genetic distances, only the 56 species shared by the phylogeny are used. The genetic distance between any pair of species is defined as the sum of the lengths of the branches on the path between the two species on the species phylogeny.

## Competing interests

The authors declare that they have no competing interests.

## Author’s contributions

All authors contribute equally. Both authors read and approved the final manuscript.

## Supplementary Material

Additional file 1 **Supplementary Material.** A pdf file compiling additional Figures and Tables referred in the text. Click here for file

Additional file 2 **Representative cases in pathway-community comparison.** A Microsoft Excel table showing pathway community overlaps with high pathway specificity or community specificity. Click here for file

Additional file 3 **Clusters of species by community structure similarity.** A Microsoft Excel table showing the clusters of species obtained by hierarchical clustering of species under the distance that equals to 1 - mutual information. Click here for file

Additional file 4 **Overview of organisms and modularities.** A Microsoft Excel table showing the list of organisms, their lifestyle classifcations and computed modularity score, both normalized and unnormalized and from four different network reconstructions. Click here for file
