# Supplementary material for: Convergent evolution of modularity in metabolic networks through different community structures
Source: BMC Evol Biol. 2012 Sep 14;12:181. doi: 10.1186/1471-2148-12-181 (PMC3534581; doi:10.1186/1471-2148-12-181)
Supplement: Additional file 1 — Supplementary Material. A pdf file compiling additional Figures and Tables referred in the text. [file 1471-2148-12-181-S1.pdf]

Convergent Evolution of Modularity in Metabolic Networks through Different Community Structures

Supplementary Information

W. Zhou and L. Nakhleh

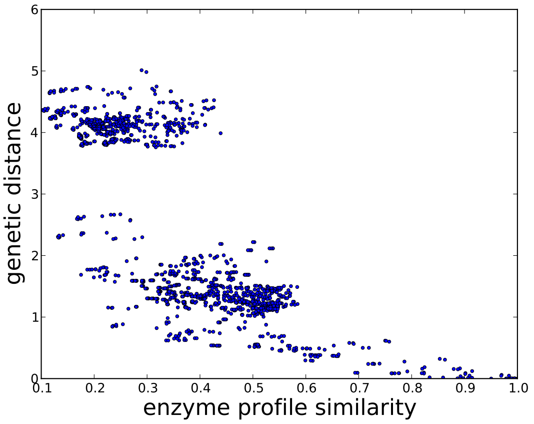

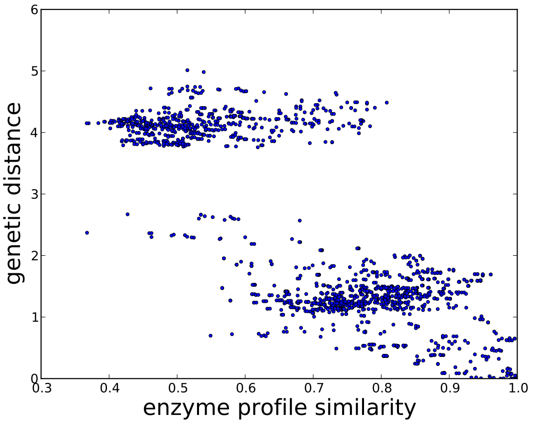

Figure 1: **Genetic Distance vs. Enzyme Profile Similarity.** (Left) Enzyme Profile Similarity is computed by the Jaccard Coefficient, i.e., $|E_1 \cap E_2|/|E_1 \cup E_2|$, with E_1 and E_2 being the enzyme profiles of the two species under comparison. (Right) Enzyme Profile Similarity is computed by, $|E_1 \cap E_2|/min(|E_1|, |E_2|)$, with E_1 and E_2 being the enzyme profiles of the two species under comparison.

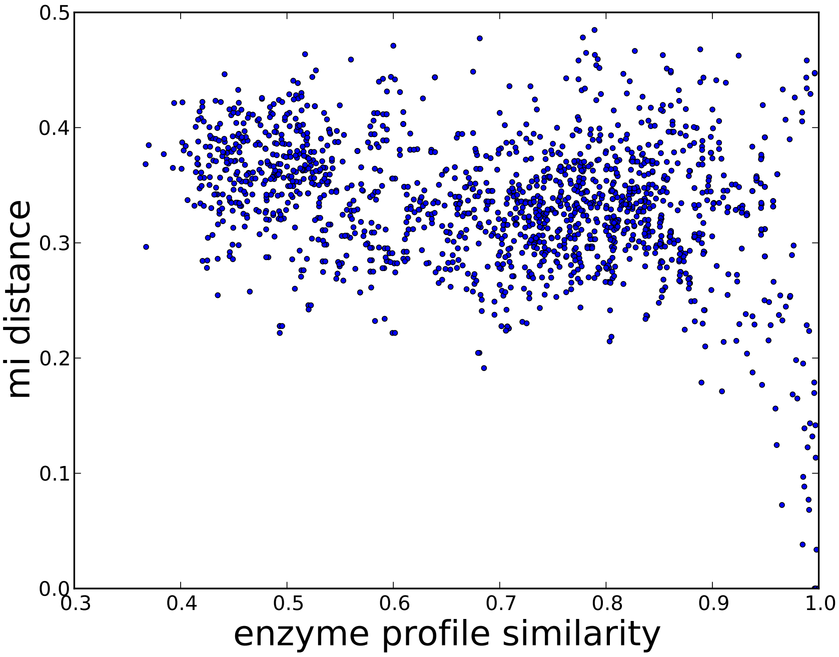

Figure 2: **Enzyme profile similarity vs. community structure difference.**, The enzyme profile similarity is negatively associated with community structure difference (Spearman's correlation coefficient $\rho = -0.26, p = 4.87 \times 10^{-27}$). When community structure difference is small, the enzyme profiles are necessarily similar. However, the reverse is not true. Similar enzyme profiles might have quite different community structures.

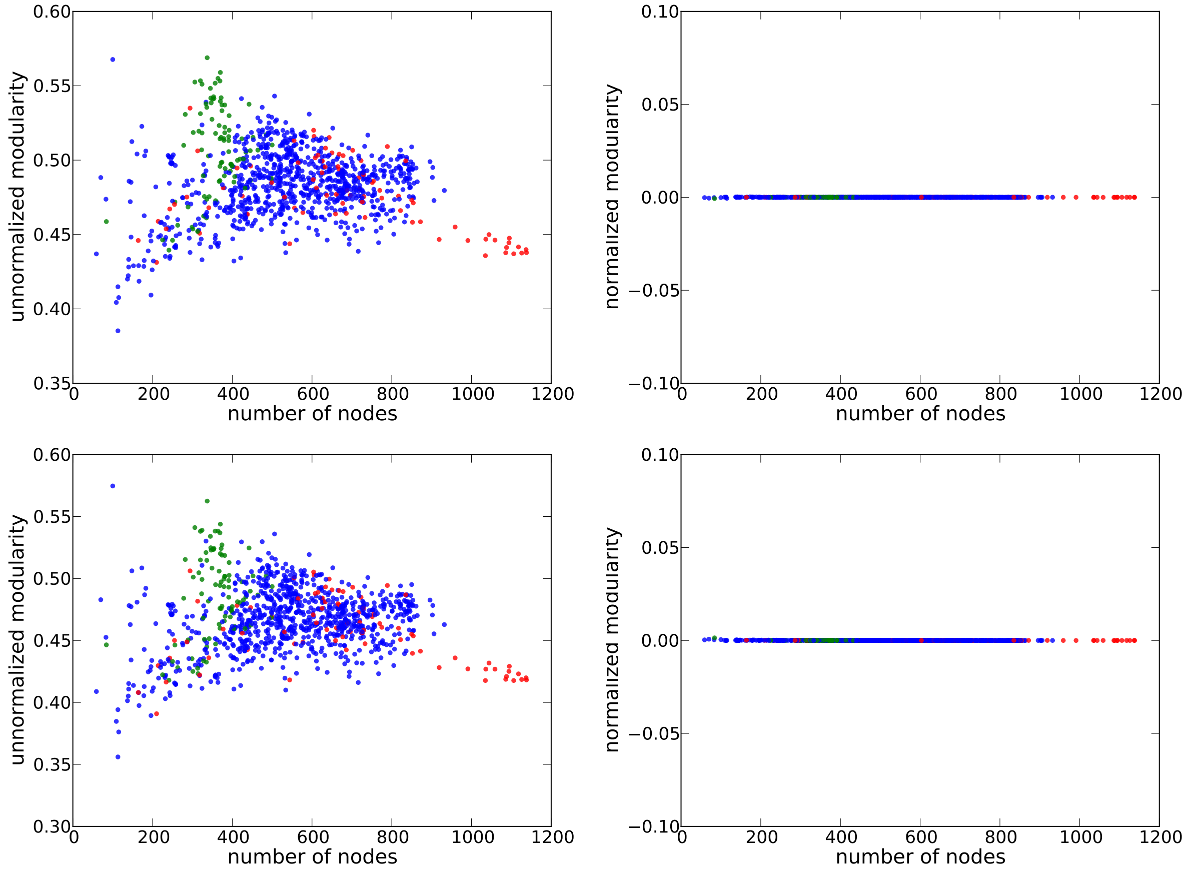

Figure 3: **Modularity vs. the number of enzymes (on rewired networks).** The dependence of modularity on the number of enzymes is gone in rewired networks. (Upper row) Random network with the same number of nodes and edges as in metabolic networks. (Bottom row) Networks obtained from rewiring metabolic networks keeping degree distribution. (Left) original modularity; (Right) normalized modularity.

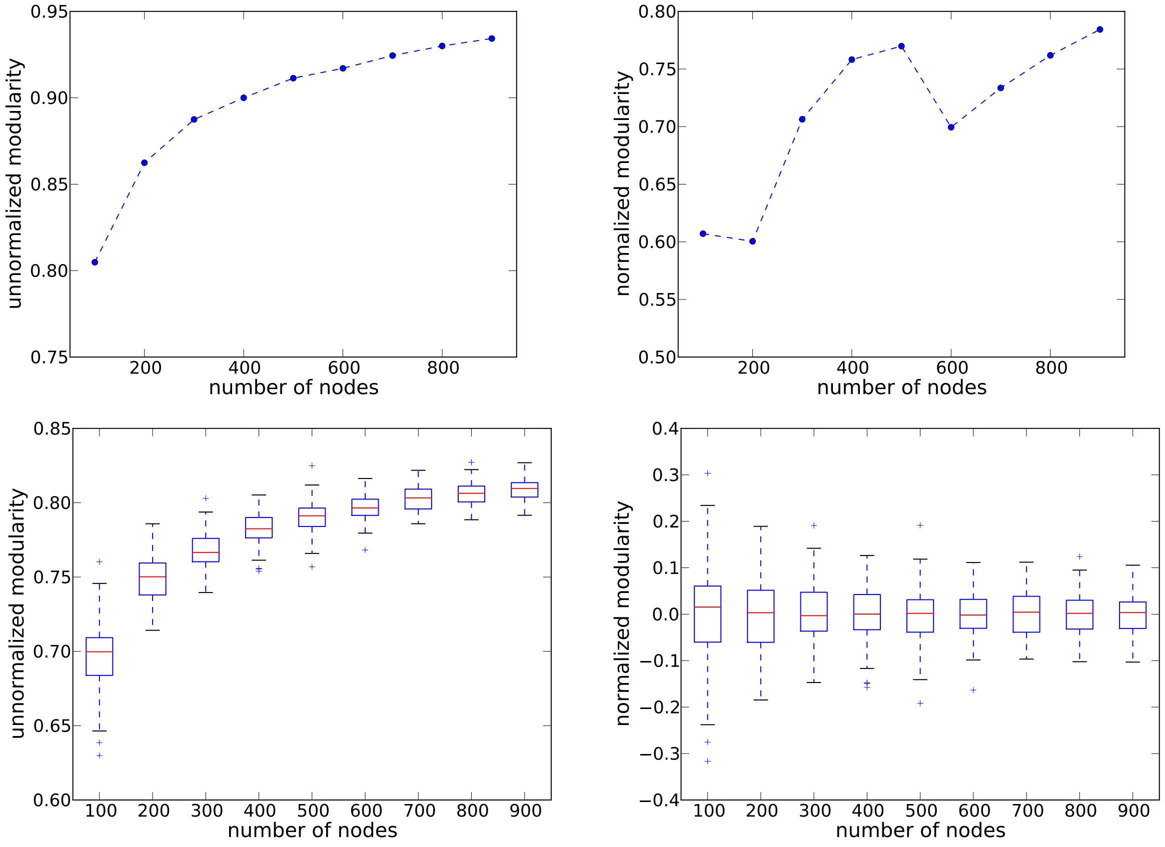

Figure 4: **Modularity vs. the number of enzymes (resemblence of linear graphs)).** (Left) original modularity; (Right) normalized modularity. (Upper row) The linear graphs. (Bottom row) Randomized linear graphs, keeping degree distribution. For each number of nodes, we compute 100 replica.

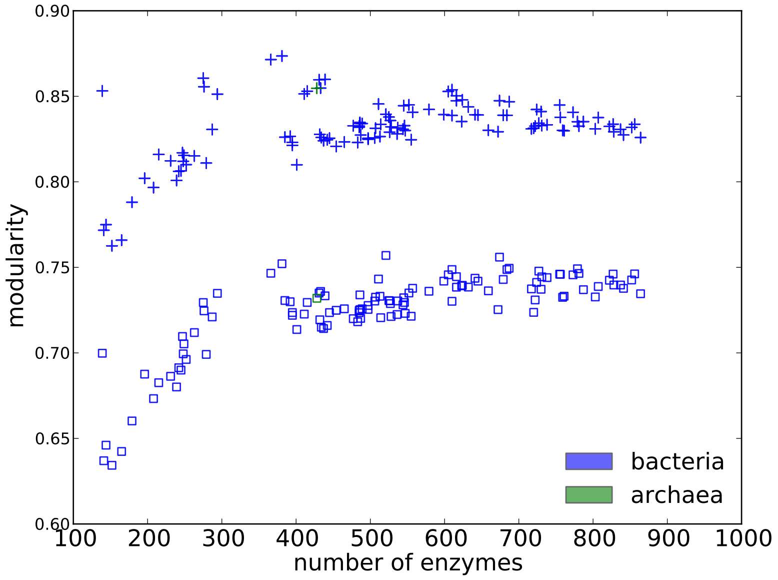

Figure 5: **Modularities vs. the number of enzymes in the line-graph transformed randomized compound networks.** Compound networks with currency deletion (of 116 microbial species) are first rewired (10000 neighbor swapping operations) and then transformed via line graph transformation [12] to approximate reaction/enzyme networks. 10 replica were made for each rewiring and the modularity scores calculated were averaged over the 10 replica of each organism. There is a positive association between modularity (both normalized and unnormalized) with the number of enzymes (Spearman's $\rho = 0.37, p = 3.06 \times 10^{-5}$ for unnormalized modularity scores and $\rho = 0.75, p = 8.97 \times 10^{-23}$ for normalized modularity scores).

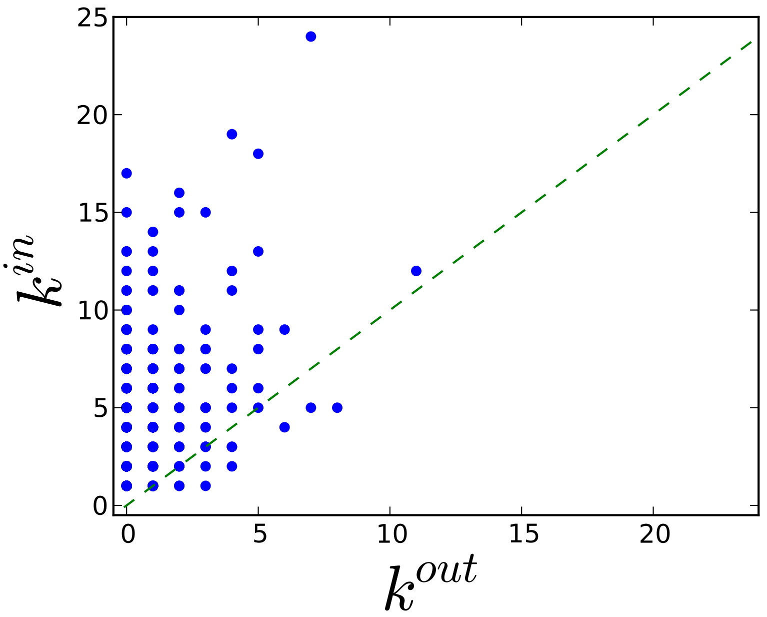

Figure 6: k^{in} **and** k^{out} **in** *E. coli*

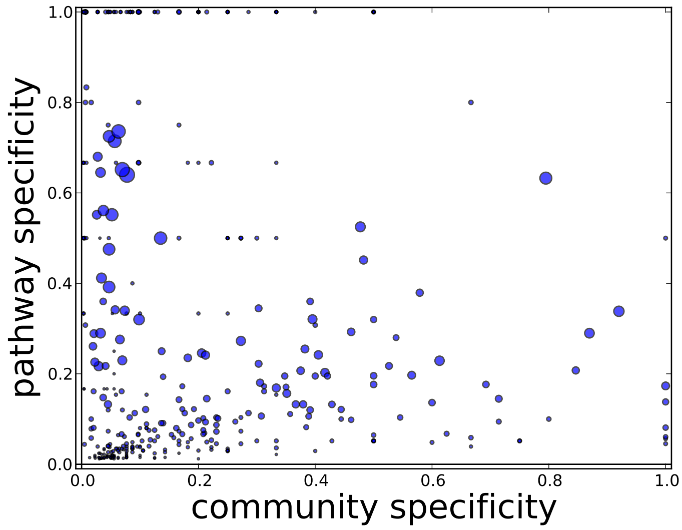

Figure 7: **Biological meaning of communities of compound network with currency deletion** The community-pathway overlap in *E. coli*'s metabolic network. Each point corresponds to a community-pathway pair, where the size of a point reflects the size of the overlap between the community and pathway. Community specificity is calculated as the fraction of pathway metabolites include in the community and the pathway specificity the fraction of community metabolites from the pathway.

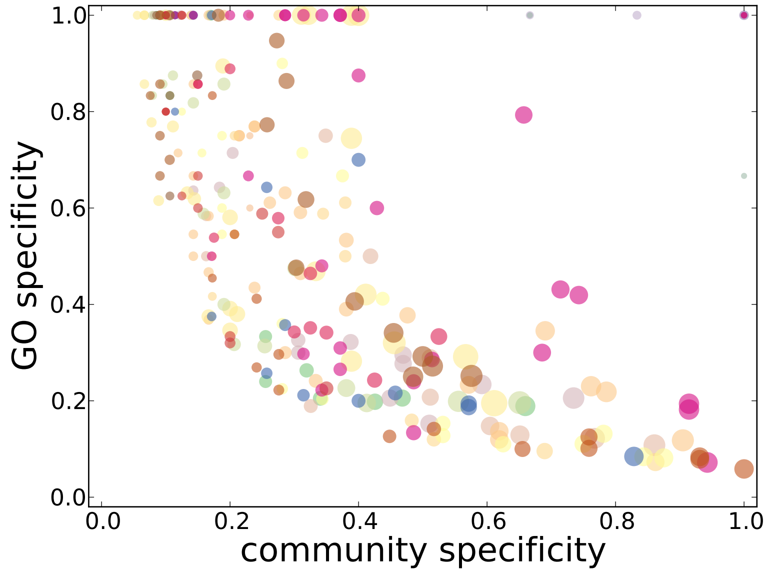

Figure 8: **GO enrichment of communities in** *E. coli* The GO term-community pairs where the GO term significantly annotates the community. The circles are made porpotional to the extent of overlap and are colored according to the community. Only GO-community pairs with a significant p-value are plotted.

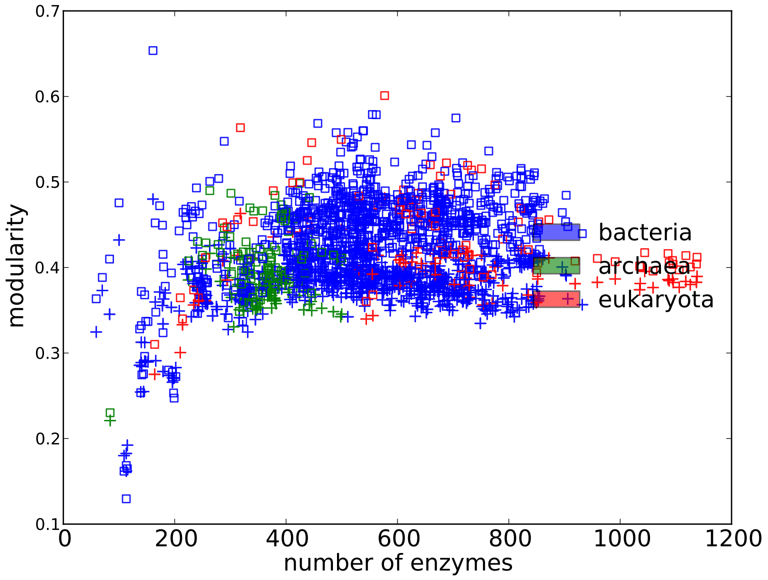

Figure 9: **Modularity vs. the number of enzymes in enzyme networks.** Modularity scores correlate slightly positively with the numbers of enzymes (Spearman's $\rho = 0.097, p = 0.0019$ for unnormalized modularity ("+" markers) and $\rho = 0.330, p = 2.37 \times 10^{-27}$ for normalized modularity (square markers)).

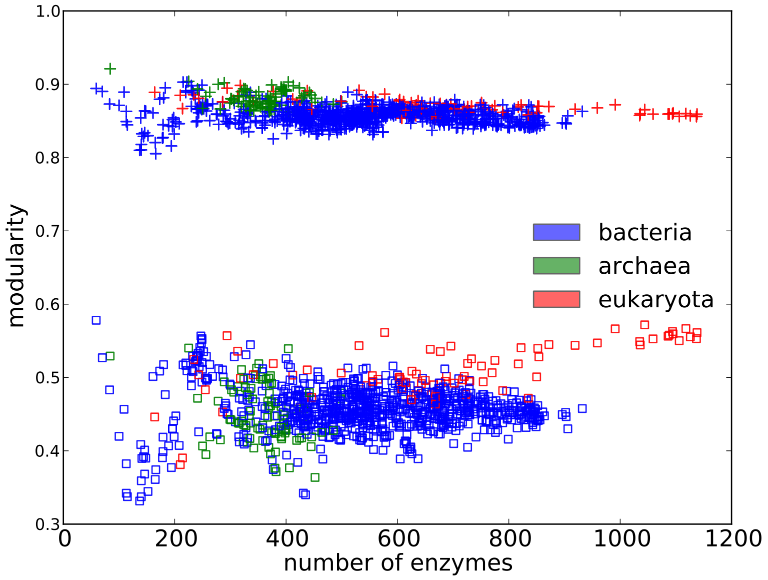

Figure 10: **Modularity vs. the number of enzymes in compound networks with currency metabolites deleted.** Unnormalized modularity scores ("+" markers) correlate slightly negatively with the numbers of enzymes (Spearman's $\rho = -0.2, p = 1.55 \times 10^{-11}$) and normalized modularity (square markers) do not correlate with the numbers of enzymes (Spearman's $\rho = 0.038, p = 0.226$).

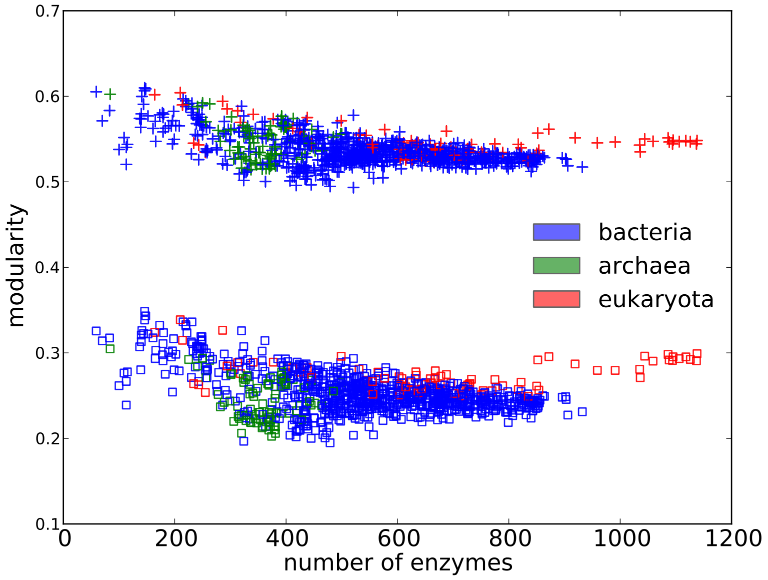

Figure 11: **Modularity vs. the number of enzymes in compound networks.** Modularity scores correlate slightly negatively with the numbers of enzymes (Spearman's $\rho = -0.387, p = 6.6 \times 10^{-38}$ for unnormalized modularity ("+" markers) and $\rho = -0.284, p = 2.38 \times 10^{-20}$ for normalized modularity (square markers)).

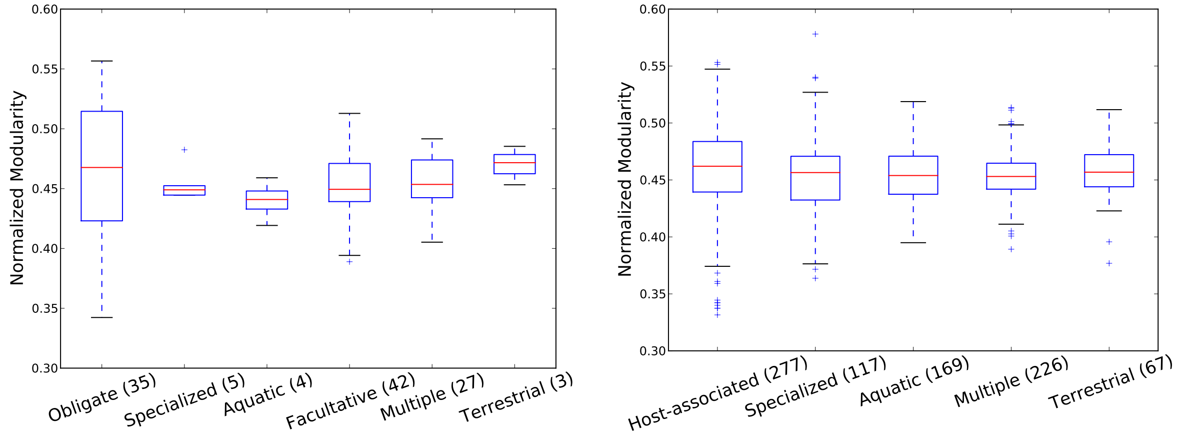

Figure 12: **Environment variability and modularity in compound networks with currency metabolites deleted.** (Left) On a small data set of 116 bacteria, habitat variability vs. normalized modularity (Kruskal-Wallis H-test $p = 0.47$) (Right) On a large data set of 806 microbes, habitat variability vs. normalized modularity (Kruskal-Wallis H-test $p = 0.016$).

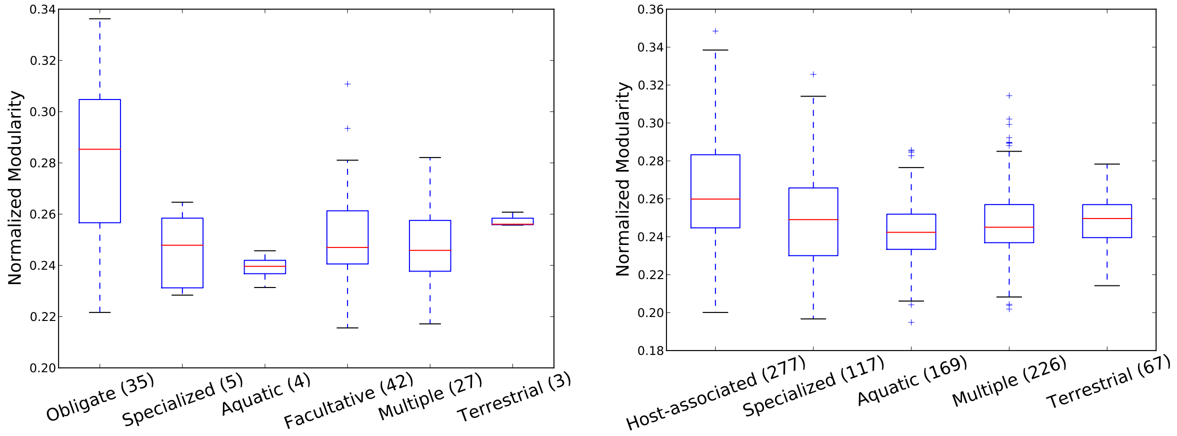

Figure 13: **Environment variability and modularity in compound networks.** (Left) On a small data set of 116 bacteria, habitat variability vs. normalized modularity (Kruskal-Wallis H-test $p = 2.55 \times 10^{-5}$) (Right) On a large data set of 806 microbes, habitat variability vs. normalized modularity (Kruskal-Wallis H-test $p = 4.12 \times 10^{-21}$).

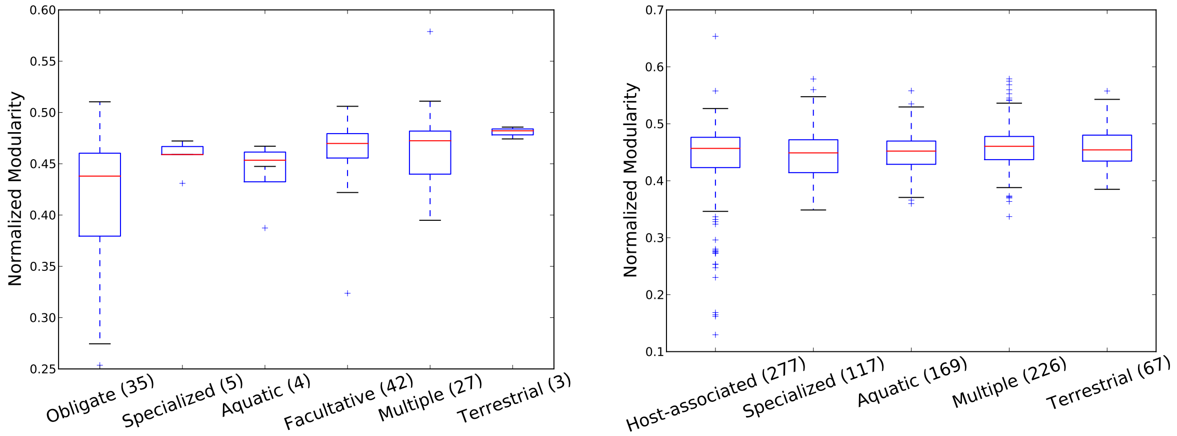

Figure 14: **Environment variability and modularity in enzyme networks.** (Left) On a small data set of 116 bacteria, habitat variability vs. normalized modularity (Kruskal-Wallis H-test $p = 0.0016$) (Right) On a large data set of 806 microbes, habitat variability vs. normalized modularity (Kruskal-Wallis H-test $p = 0.0148$).

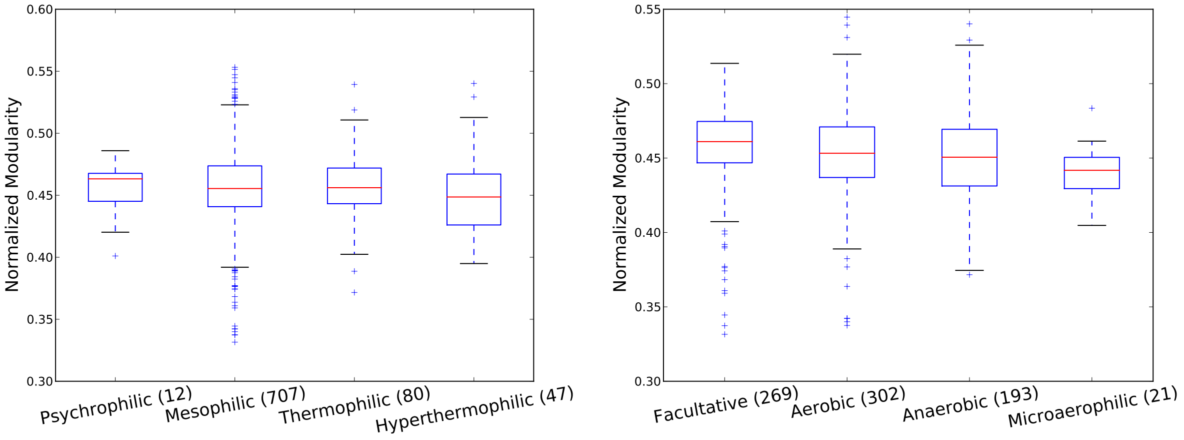

Figure 15: **Modularity and environment factors in compound networks with currency metabolites deleted.** (Left) normalized modularity vs. oxygen requirement (Kruskal-Wallis H-test $p = 0.35$); (Right) normalized modularity vs. temperature requirement (Kruskal-Wallis H-test $p = 6.15 \times 10^{-5}$).

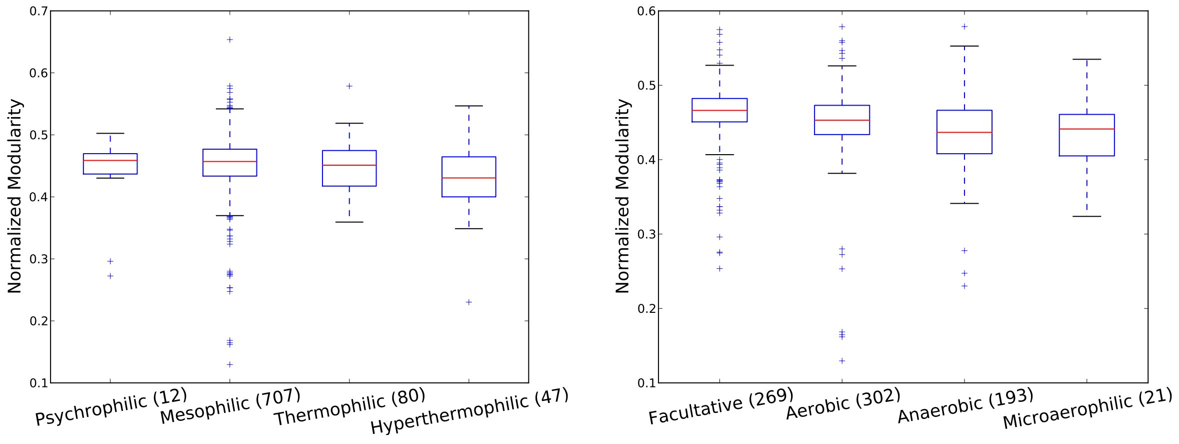

Figure 16: **Modularity and environment factors in enzyme networks.** (Left) normalized modularity vs. oxygen requirement (Kruskal-Wallis H-test $p = 0.02$); (Right) normalized modularity vs. temperature requirement (Kruskal-Wallis H-test $p = 2.33 \times 10^{-12}$).

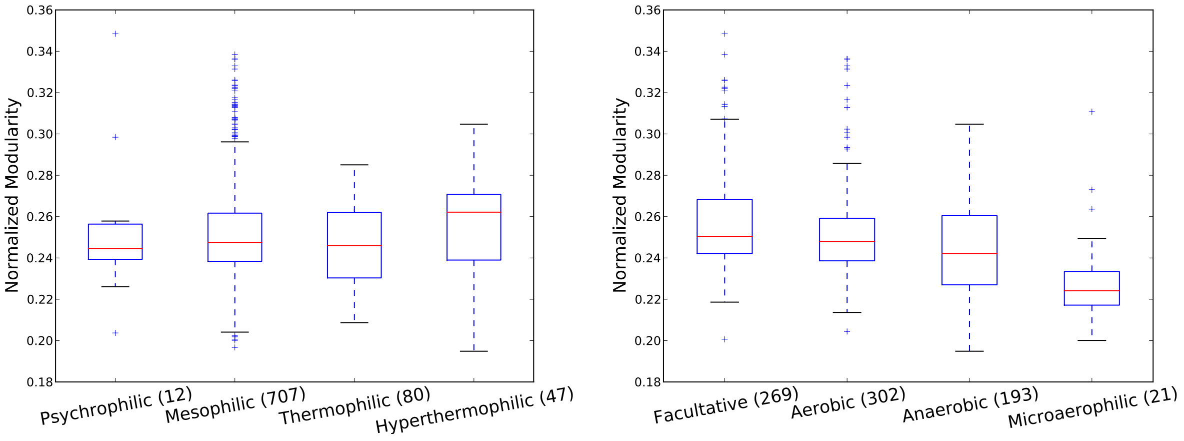

Figure 17: **Modularity and environment factors in compound networks.** (Left) normalized modularity vs. oxygen requirement (Kruskal-Wallis H-test $p = 0.047$); (Right) normalized modularity vs. temperature requirement (Kruskal-Wallis H-test $p = 2.52 \times 10^{-11}$).

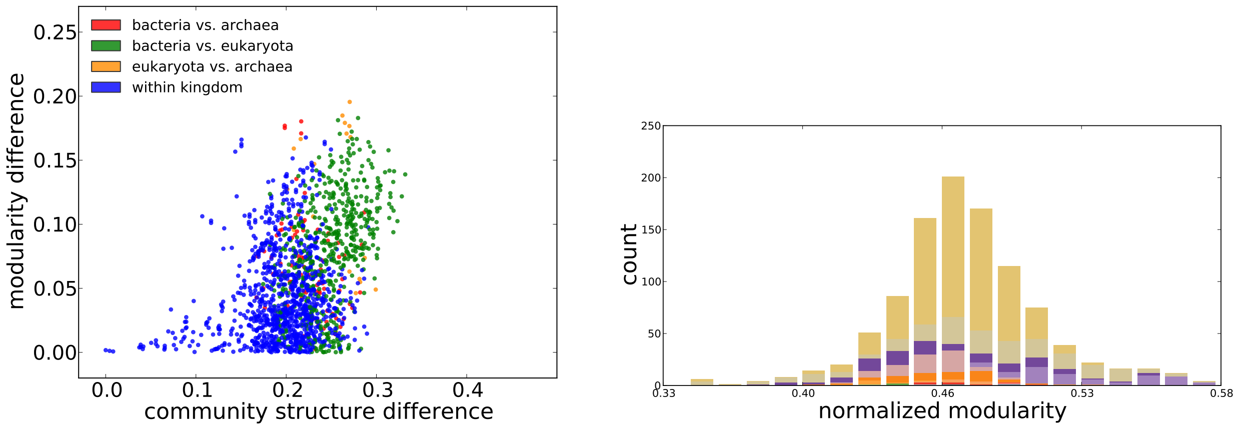

Figure 18: **Community structure vs. modularity in compound networks with currency metabolites deleted.** Left) Community structure difference vs. modularity difference: Difference in community structure is computed by $1 - MI$ where MI is the mutual information between the two community structures. Right) Distribution of modularity scores colored by the cluster to which the community structures of the metabolic network belongs (See Methods for the method used to cluster species based on the distance in the community structures). Modularity scores are normalized with respect to scores based on randomized networks (See Methods). The normalized modularity is believed to have network size-dependent factors removed, allowing networks of different sizes and connectivity to be comparable in modularity [1]. Each color corresponds to a community structure cluster. The height of the bar (or bar segment) is proportional to the number of species in each cluster falling into the particular bin of modularity scores.

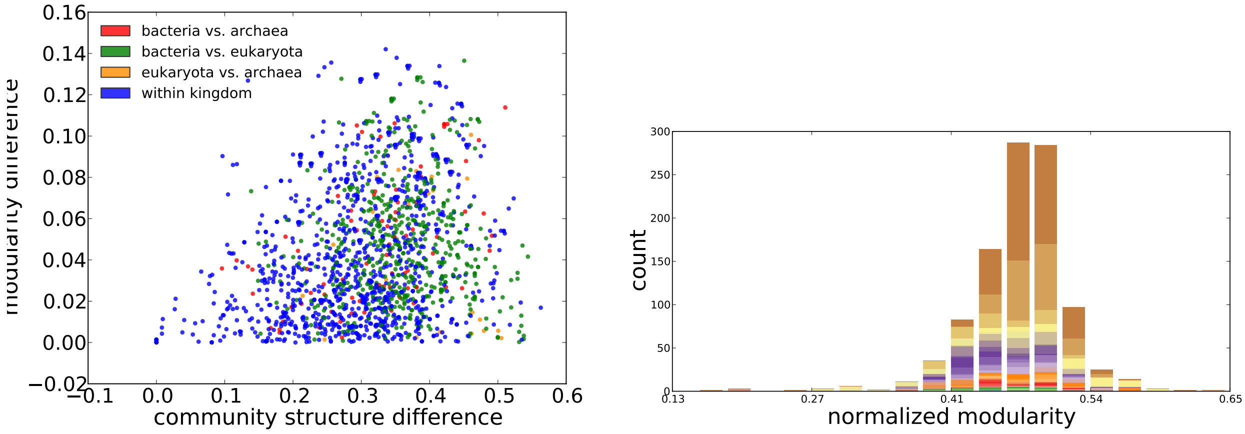

Figure 19: **Community structure vs. modularity in enzyme networks.** Left) Community structure difference vs. modularity difference: Difference in community structure is computed by $1 - MI$ where MI is the mutual information between the two community structures. Right) Distribution of modularity scores colored by the cluster to which the community structures of the metabolic network belongs (See Methods for the method used to cluster species based on the distance in the community structures). Modularity scores are normalized with respect to scores based on randomized networks (See Methods). The normalized modularity is believed to have network size-dependent factors removed, allowing networks of different sizes and connectivity to be comparable in modularity [1]. Each color corresponds to a community structure cluster. The height of the bar (or bar segment) is proportional to the number of species in each cluster falling into the particular bin of modularity scores.

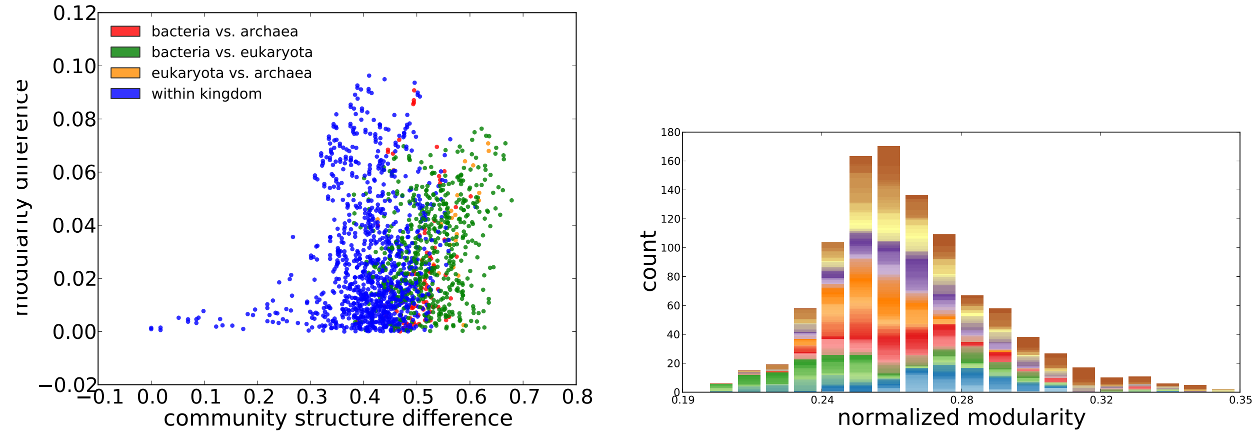

Figure 20: **Community structure vs. modularity in compound networks.** Left) Community structure difference vs. modularity difference: Difference in community structure is computed by $1 - MI$ where MI is the mutual information between the two community structures. Right) Distribution of modularity scores colored by the cluster to which the community structures of the metabolic network belongs (See Methods for the method used to cluster species based on the distance in the community structures). Modularity scores are normalized with respect to scores based on randomized networks (See Methods). The normalized modularity is believed to have network size-dependent factors removed, allowing networks of different sizes and connectivity to be comparable in modularity [1]. Each color corresponds to a community structure cluster. The height of the bar (or bar segment) is proportional to the number of species in each cluster falling into the particular bin of modularity scores.

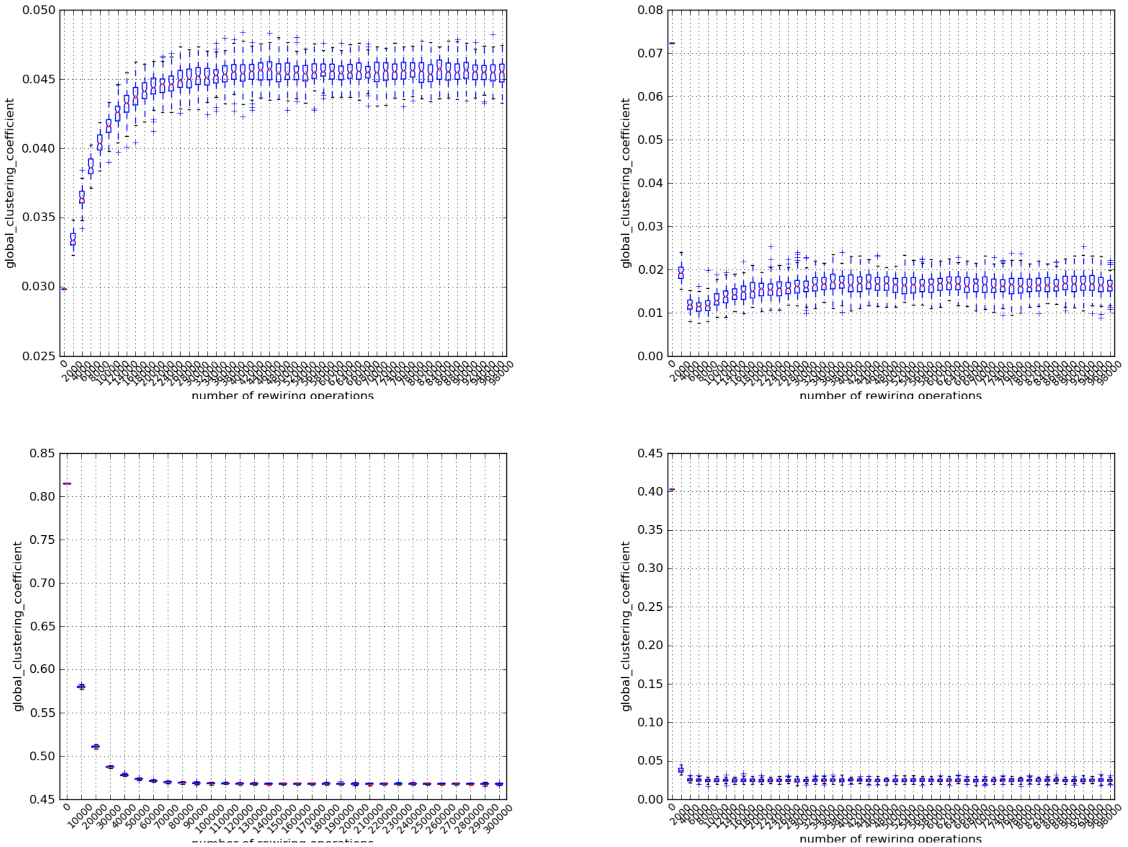

Figure 21: **Convergence of rewiring.** Convergence of rewiring is measured in the leveling of global clustering coefficient in the largest network compared among different species. Networks of four semantics (see methods for abbreviations) are shown: (Upper left) Compound network. The number of rewiring operations, as determined by the human network, is set to 20000; (Upper right) Compound network with currency metabolites deleted. The number of rewiring operations, as determined by the human network, is set to 40000; (Lower left) Enzyme network. The number of rewiring operations, as determined by the mouse network, is set to 80000; (Lower right) Enzyme network with currency link deleted. The number of rewiring operations, as determined by the mouse network, is set to 8000.

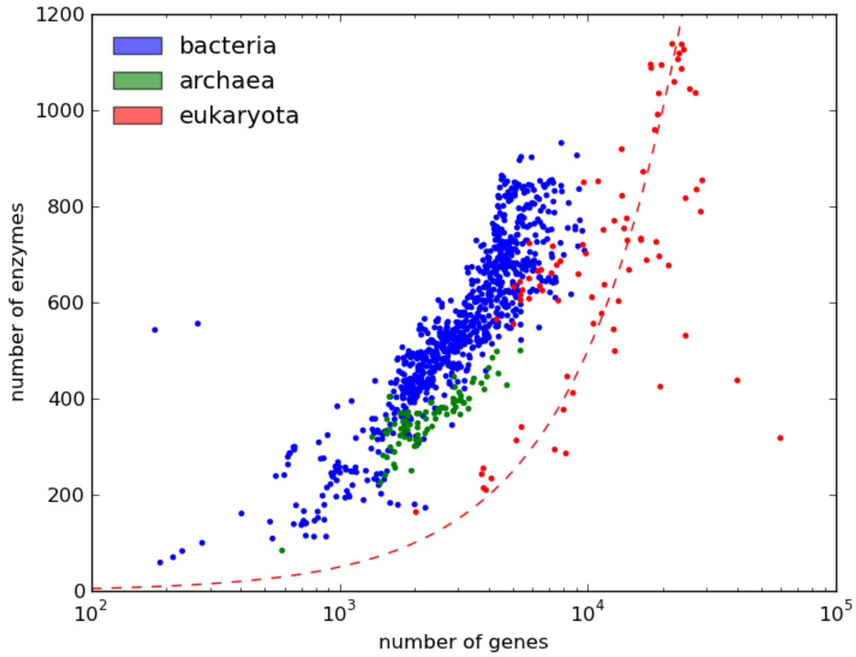

Figure 22: **Summary of gene and enzymatic annotation.** Dashed line corresponds to $y = 0.05x$ where the number of annotated enzymes is 20% the all number of annotated genes.

group 1	group 2	one-tail Wilcoxon rank-sum statistic	p-value	significant
Psychrophilic	Mesophilic	3082.0	0.120	no
Psychrophilic	Thermophilic	252.0	0.0125	no
Psychrophilic	Hyperthermophilic	102.0	1.17e-3	yes
Mesophilic	Thermophilic	21060.0	1.77e-4	yes
Mesophilic	Hyperthermophilic	8711.0	6.73e-08	yes
Thermophilic	Hyperthermophilic	1201.0	8.14e-4	yes

Table 1: **Pairwise comparison between different categories of temperature requirements.** Significance are evaluated after Bonferroni correction for multiple testing.

Table 2: **Comparison of the Q scores computed by various algorithms on benchmark data sets.** Algorithms: "GN" is the method of [2]; "FG" is the method of [3]; "DA" is the method of [4]; "LR" is the method of [5]; "VP" and "LP" are vector programming and linear programming based algorithms by [6]; "A1" is Algorithm 1 (see Main Text). Algorithms that are slow on very large networks are labeled 'NA', as in [6].

Benchmark data set		modularity Q						
	#nodes	GN	FG	DA	LR	VP	LP	A1
karate club [7]	34	0.401	0.381	0.419	0.419	0.420	0.420	0.420
jazz musicians [8]	198	0.405	0.439	0.445	0.442	0.445	0.445	0.445
C. elegans [9]	453	0.403	0.402	0.434	0.435	0.450	NA	0.453
E-mail [10]	1,133	0.532	0.494	0.574	0.572	0.579	NA	0.581
key signing [11]	10,680	0.816	0.733	0.846	0.855	NA	NA	0.874

Table 3: **Time spent for computing community structure on benchmark data.**

Benchmark data set	#nodes	wall time (s)
karate club [7]	34	0.014 sec
jazz musicians [8]	198	1.092 sec
C. elegans [9]	453	5.19 sec
E-mail [10]	1,133	34.193 sec
key signing [11]	10,680	42 min

References

[1] N. Kashtan and U. Alon, “Spontaneous evolution of modularity and network motifs.” *Proceedings of the National Academy of Sciences of the United States of America*, vol. 102, no. 39, pp. 13 773–8, Sep. 2005. [Online]. Available: http://www.pnas.org/content/102/39/13773.abstract http://www.pubmedcentral.nih.gov/articlerender.fcgi?artid=1236541&tool=pmcentrez&rendertype=abst

[2] M. Newman and M. Girvan, “Finding and evaluating community structure in networks,” *Physical Review E*, vol. 69, no. 2, p. 26113, Feb. 2004. [Online]. Available: http://link.aps.org/abstract/PRE/v69/e026113 http://link.aps.org/doi/10.1103/PhysRevE.69.026113

[3] A. Clauset, M. E. J. Newman, and C. Moore, “Finding community structure in very large networks,” *Physical Review E*, vol. 70, no. 6, p. 66111, Dec. 2004. [Online]. Available: http://link.aps.org/abstract/PRE/v70/e066111

[4] J. Duch and A. Arenas, “Community detection in complex networks using extremal optimization,” *Physical Review E*, vol. 72, no. 2, p. 27104, Aug. 2005. [Online]. Available: http://prola.aps.org/abstract/PRE/v72/i2/e027104 http://link.aps.org/doi/10.1103/PhysRevE.72.027104

[5] M. E. J. Newman, “Modularity and community structure in networks,” *Proceedings of the National Academy of Sciences*, vol. 103, no. 23, pp. 8577–8582, Jun. 2006. [Online]. Available: http://www.pnas.org/content/103/23/8577.abstract

[6] G. Agarwal and D. Kempe, “Modularity-maximizing graph communities via mathematical programming,” *Eur. Phys. J. B*, vol. 66, no. 3, pp. 409–418, Dec. 2008. [Online]. Available: http://dx.doi.org/10.1140/epjb/e2008-00425-1

[7] W. W. Zachary, “An information flow model for conflict and fission in small groups,” *J Anthropol Res*, vol. 33, pp. 452–473, 1970.

[8] P. Gleiser and L. Danon, “Community Structure in Jazz,” *Advances in Complex Systems*, vol. 6, no. 4, p. 12, Jul. 2003. [Online]. Available: http://arxiv.org/abs/cond-mat/0307434

[9] H. Jeong, B. Tombor, R. Albert, Z. N. Oltvai, and A.-L. Barabási, “The large-scale organization of metabolic networks,” *Nature*, vol. 407, no. 6804, pp. 651–654, Oct. 2000. [Online]. Available: http://dx.doi.org/10.1038/35036627

[10] R. Guimerà, L. Danon, A. Díaz-Guilera, F. Giralt, and A. Arenas, “Self-similar community structure in a network of human interactions,” *Physical Review E*, vol. 68, no. 6, Dec. 2003. [Online]. Available: http://pre.aps.org/abstract/PRE/v68/i6/e065103

[11] X. Guardiola, R. Guimera, A. Arenas, A. Diaz-Guilera, D. Streib, and L. A. N. Amaral, “arXivcondmat0206240,” *Condensed Matter: Disordered Systems and Neural Networks*, Jun. 2002. [Online]. Available: http://xxx.lanl.gov/abs/cond-mat/0206240 http://arxiv.org/abs/cond-mat/0206240

[12] J. C. Nacher, N. Ueda, T. Yamada, M. Kanehisa, and T. Akutsu, “Clustering under the line graph transformation: application to reaction network.” *BMC bioinformatics*, vol. 5, p. 207, Jan. 2004. [Online]. Available: http://www.pubmedcentral.nih.gov/articlerender.fcgi?artid=545960&tool=pmcentrez&rendertype=abstr
